# Novel Triple Stimuli Responsive Interpenetrating Poly(Carboxybetaine Methacrylate)/Poly(Sulfobetaine Methacrylate) Network

**DOI:** 10.3390/gels9020090

**Published:** 2023-01-20

**Authors:** Konstans Ruseva, Katerina Todorova, Tanya Zhivkova, Rositsa Milcheva, Dimitar Ivanov, Petar Dimitrov, Radostina Alexandrova, Elena Vassileva

**Affiliations:** 1 Laboratory on Structure and Properties of Polymers, Department of Pharmaceutical and Applied Organic Chemistry, Faculty of Chemistry and Pharmacy, Sofia University “St. Kl. Ohridski”, 1 J. Bourchier Blvd., 1164 Sofia, Bulgaria; 2 Institute of Experimental Morphology, Pathology and Anthropology with Museum, Bulgarian Academy of Sciences, Acad. G. Bonchev Str., Bl. 25, 1113 Sofia, Bulgaria

**Keywords:** interpenetrating polymer network, poly(sulfobetaine methacrylate), poly(carboxybetaine methacrylate), triple stimuli responsiveness, biomaterial

## Abstract

The study reports the synthesis and characterization of novel triple stimuli responsive interpenetrating polymer network (IPN) based on two polyzwitterionic networks, namely of poly(carboxybetaine methacrylate) and poly(sulfobetaine methacrylate). The zwitterionic IPN hydrogel demonstrates the ability to expand or shrink in response to changes in three “biological” external stimuli such as temperature, pH, and salt concentration. The IPN hydrogel shows good mechanical stability. In addition, other important features such as non-cytotoxicity and antibiofouling activity against three widespread bacteria as *P. Aeruginosa*, *A. Baumanii,* and *K. Pneumoniae* are demonstrated. The in vivo behavior of the novel zwitterionic IPN hydrogel suggests that this smart material has very good potential as a biomaterial.

## 1. Introduction

Hydrogels are three-dimensional networks made from crosslinked polymers bearing in their side chains hydrophilic functional groups such as –COOH, –OH, –CONH_2_, -SO_3_H, R_4_N^+^, etc. These polymer networks are able to absorb and retain high water amounts, much higher than their own weight, which results in a significant increase in their volume upon swelling. The functional groups define the hydrogels’ responsiveness to changes in the chemical, physical or biological environment which leads to a dramatic change in the hydrogels’ size and shape. Thus, hydrogels could undergo swelling or deswelling in response to external stimuli which defines them as “smart” materials. Some of the external stimuli that could influence the hydrogels’ behavior are temperature, pH, and salt concentration [1]. The hydrogels that respond to more than one external stimulus are rarely reported although they currently find an increasing number of different applications [2].

Hydrogels are soft, flexible, and usually biocompatible materials that mimic the mechanical performance of biological tissues as well as provide a good environment for cell survival and proliferation [3]. Hence, they have many biomedical applications, e.g., in tissue engineering, drug delivery, wound healing, immunotherapy, plastic surgery, etc. [3]. Smart hydrogels are also commonly used as biosensors for enzyme immobilization [4], bio actuators [5], draw agents in osmosis desalination [6], 3D and 4D printing, etc. However, the broad spectrum of hydrogel applications is somehow limited by their weak mechanical properties [7]. For this reason, many efforts are underway to improve the mechanical behavior of hydrogels. Some of the strategies for strengthening hydrogels used so far include the formation of polymer nanocomposites [8], polymer hydrogels with ideally homogeneous networks [9], fiber-reinforced hydrogels [10] and interpenetrating polymer networks (IPNs) [11]. The IPN approach is a successful method for strengthening the hydrogel structure via the mutual interlacing of two polymer networks, thus resulting in one of the toughest and strongest hydrogels obtained so far, the so-called double networks [11] which are a special case of IPNs. IPNs could be obtained either via the simultaneous formation of both polymer networks or via the sequential method where the 2nd network is obtained in situ in the previously obtained 1st network [12]. In addition to the good mechanical stability, IPNs’ approach also provides a tool to control the properties of the hydrogels by proper choice of their constituents thus allowing the combination of different functionalities and behavior in one material.

Polyzwitterions (PZs) are non-toxic hydrophilic polymers with unique properties defined by their zwitterionic structure. They bear an equal number of negatively and positively charged groups in their side chains keeping overall neutrality. The presence of at least two functional groups in each side chain ensures the PZs’ ability to strongly bind high amounts of water molecules. Depending on the type of anionic group they possess, the three most widespread PZs types are defined—polysulfobetaines (PSB), polycarboxybetaines (PCB), and polyphosphobetaines (PPhB). Many studies have shown that PSB and PCB-based hydrogels are known to possess outstanding antifouling properties as they resist non-specific protein adsorption as well as cell adhesion [13,14]. Their non-cytotoxicity, in combination with good antibiofouling ability, makes PZs attractive materials for biomedical applications. Due to their functional groups, PZs can “intelligently” respond to various external stimuli by changing their volume, shape, and size. PSBs are known to be temperature-responsive with upper critical solution temperature behavior [15], while PCB respond to pH changes due to their -COOH groups [16]. In addition, both polymers (PSB and PCB) exhibit an antipolyelectrolyte behavior, i.e., they expand in presence of salt as opposed to polyelectrolytes which are known to shrink in salt aqueous solutions [16]. There are studies reporting the salt-responsiveness either of PSB [17,18] or PCB [19] as well as the pH- responsiveness of PCB [19] and the temperature responsiveness of PSB, but no attempt was made so far to study their triple stimuli responsiveness provided by the combination of PSB and PCB in one material.

The aim of the current study is to obtain triple stimuli-responsive hydrogels, simultaneously sensitive to changes in temperature, pH, and salt concentration via the synthesis of PCB/PSB IPN. This material is also expected to be non-cytotoxic and biocompatible due to the inherent biocompatibility of both IPN constituents—PSB and PCB [20,21,22]. The triple stimuli smart response of the obtained PCB/PSB IPN as well as its ability to bind water strongly and prevent bacterial adhesion on its surface is also studied. The in vivo biocompatibility of the developed novel PCB/PSB IPN is demonstrated.

## 2. Results and Discussion

### 2.1. Microhardness

**Scheme 1:**
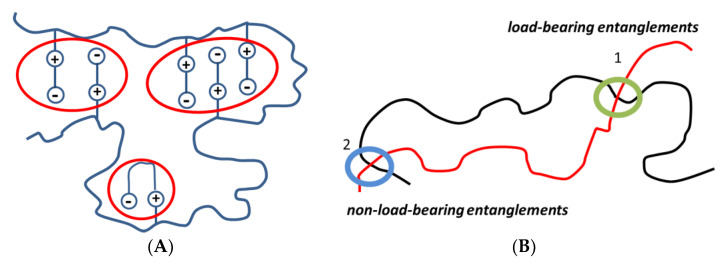
(**A**) Dipole-dipole zip clusters formed in single PSB and PCB networks. (**B**) load-bearing (**1**) and non-load-bearing (**2**) entanglements between PSB (black line) and PCB (red line) chains in their IPN.

The microhardness of the PCB/PSB IPN network was determined and compared to the microhardness of the single PSB and PCB networks (Table 1). The PCB/PSB IPN has lower microhardness as compared to the single PCB network. At the same time, IPN’s microhardness is comparable, although a little bit lower, to the microhardness of the single PSB network. This was not fully expected as IPNs are known to form chain-interlaced structures which should define higher microhardness as compared to the single networks, due to the formed chain entanglements. The low microhardness value of the PCB/PSB IPN could be explained by two factors, namely: (i) the disturbed physical network, formed via dipole-dipole interactions between the zwitterionic side chain moieties (Scheme 1A) in the IPN as compared to the single polymer networks. Both neat polymers, PSB and PCB, are known to form dipole-dipole zip clusters (Scheme 1A), which play the role of physical network junctions, formed additionally to the PEGDA chemical crosslinking. The interlacing between both zwitterionic networks in their IPN hampers the formation of these clusters, thus decreasing the amount of the physical junctions as compared to the neat PSB and PCB, respectively lowering the IPNs microhardness; and (ii) the fact that not all chain entanglements are able to carry substantial load upon the network deformation.

The chain entanglements could be divided into two groups: (i) load-bearing entanglements (designated with 1 in Scheme 1B), which are usually formed by the interlacing of two long polymer chains and this interlacing takes place far from the chain ends as well as (ii) non-load-bearing entanglements (designated with 2 in Scheme 1B), which are the entanglements between both polymers chains formed close to the chains ends. The in situ formation of the 2nd network in the presence of the 1st one during the IPN formation is expected to generate a less ideal network, i.e., containing more chain ends, and thus the number of the non-load-bearing entanglements is expected to be higher in the IPN obtained via the sequential method. The microhardness for such a “defects-enriched” network could be expected to be lower due to the increased number of chain ends, respectively to the number of non-load bearing entanglements.

It could be concluded that the PCB/PSB IPN network microhardness is comparable to the microhardness of other polymeric materials and shows that the newly developed material is strong enough to be used as biomaterial.

### 2.2. Rheology

**Figure 1:**
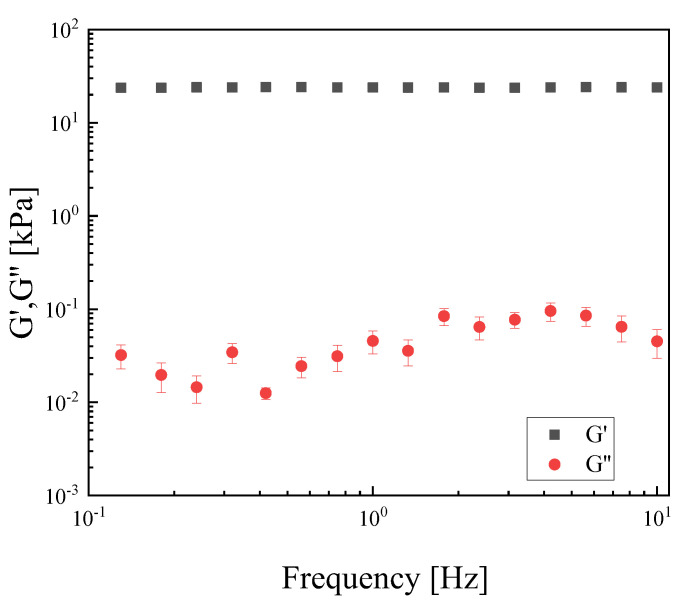
Storage (G′) and loss (G″) moduli of PCB/PSB IPN hydrogel dependence on frequency.

The viscoelastic properties of PCB/PSB IPN hydrogel were studied via frequency sweep of the storage (G′) and loss (G″) moduli (Figure 1). The storage modulus of the PCB/PSB IPN (G′~ 23.5 kPa) is much higher than the loss modulus (G″~0.5 kPa) within the entire frequency range from 0.1 to 10 Hz, indicating that the IPN hydrogel is mechanically stable. When comparing G′ modulus of the IPN hydrogel to the G′ moduli of both single PSB [18] and PCB [19] hydrogels, obtained using the same or similar crosslinking agent concentrations, it appeared that the IPN hydrogels are much stronger. For example, while the 4PCB hydrogel has G′~15 kPa [19] and PSB hydrogel with comparable crosslinking density (3PSB) has G′~ 6 kPa [18], the PCB/PSB IPN hydrogel has much higher G′ value (G′~ 23.5 kPa). This different rheological behavior of the IPN hydrogel could be related to the entanglements obtained via the mutual interlacing of both PSB and PCB networks into the IPN, which enhances the IPN’s storage modulus. Thus, the PCB/PSB IPN hydrogel has improved mechanical strength as compared to its constituent single networks, which makes it very appropriate to be used in biomedical applications.

It should be mentioned here that the different roles that the chain entanglements in the IPN play for the microhardness and the storage modulus could originate from the fact that the former is measured for dry IPN sample, while the latter is obtained for IPN hydrogel at its equilibrium swelling ratio.

### 2.3. Temperature Responsiveness of PCB/PSB IPN Hydrogel

**Figure 2:**
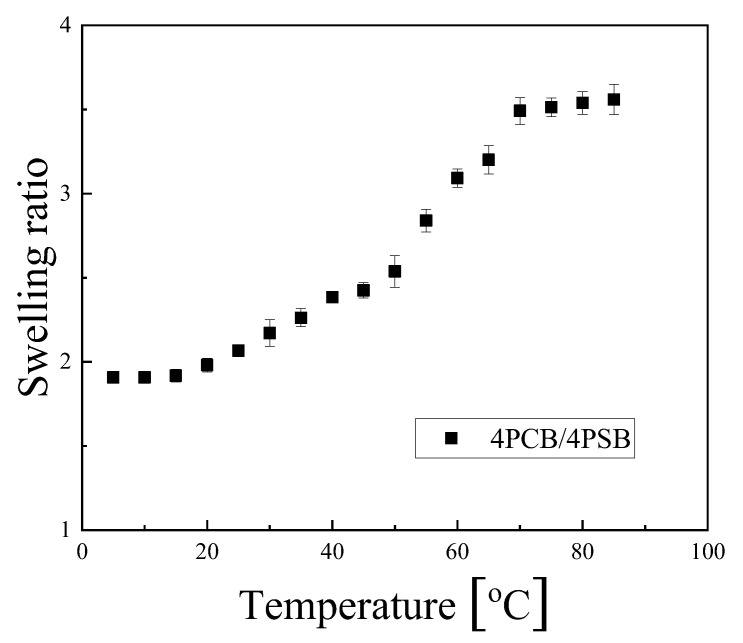
Temperature responsiveness of PCB/PSB IPN hydrogel studied in distilled water (pH = 6.4): swelling for 8 h was performed at a defined temperature within the temperature range from 5 to 90 °C.

**Scheme 2:**
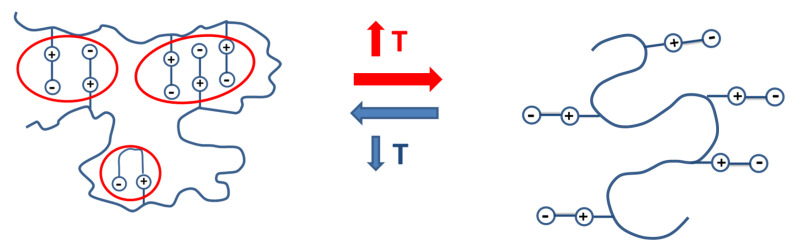
Temperature increase causes disruption of the PSB physical network and hence the hydrogel expansion.

As PSB is a temperature-responsive polymer, it was interesting to check if it imparts this smart behavior also to the PCB/PSB IPN as PCB is known not to respond to temperature changes. The PCB/PSB IPN hydrogel exhibits a temperature-dependent swelling profile as demonstrated in Figure 2. Its swelling ratio (SR) increases almost twice (from 1.8 to 3.5) upon temperature increase in the studied temperature range. In a previous study, we have demonstrated that PSB hydrogels show upper critical solution temperature behavior, i.e., their swelling is enhanced upon heating, and the magnitude of this swelling enhancement is defined mainly by the crosslinking agent poly(ethylene glycol) diacrylate (PEGDA) amount: the lower the crosslinking agent concentration, the stronger the SR increase with temperature is [18]. Similarly to the observed for the PCB/PSB IPN behavior, at low temperatures (T < 20 °C) the PSB hydrogels shrink due to the formation of the dipole-dipole zip clusters between the PSB side chain zwitterionic moieties (Scheme 1A). The gradual temperature increase from 20 °C to 70 °C results in destroying the dipole-dipole zwitterionic clusters (Scheme 2), and hence the PCB/PSB IPN hydrogel expands, reaching a plateau at T > 70 °C. This plateau is defined by the constraints that the chemical crosslinking with PEGDA imparts when the entire physical network of PSB is destroyed upon heating.

Thus, the PSB component of the IPN successfully imparts temperature responsiveness to the PCB/PSB IPN making it temperature responsive material.

### 2.4. pH Responsiveness of PCB/PSB IPN Hydrogel

**Figure 3:**
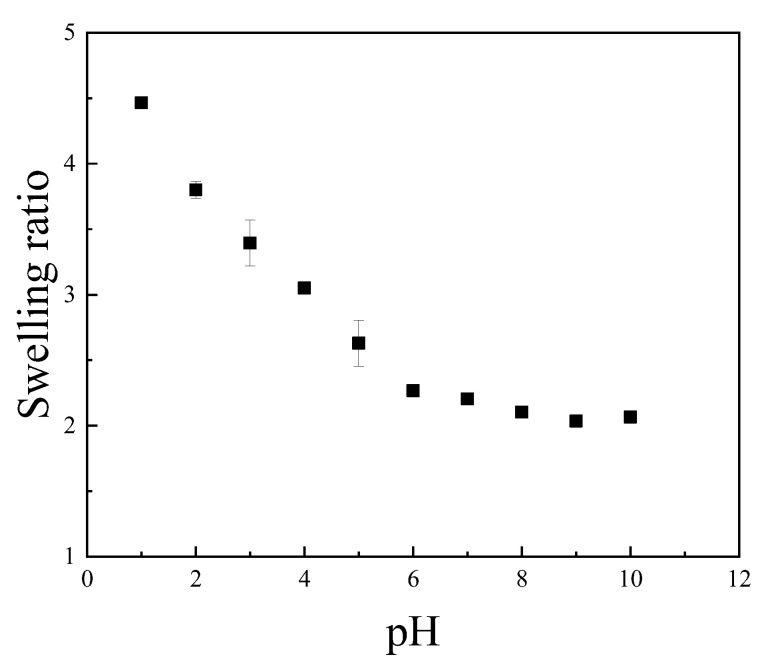
pH responsiveness of PCB/PSB IPN hydrogel at 25 °C in distilled water.

Due to the presence of –COOH groups in its side chains, PCB is known to be pH responsive polymer. Thus, the influence of pH on the PCB/PSB IPN hydrogel swelling was studied (Figure 3). In an acidic medium, below pH~4.5–5, PCB exists as polycation due to the positively charged quaternary groups in its side chains (–COOH groups are protonated below their pKa~4.5–5, thus transforming the zwitterionic moieties into cationic ones). Repulsive electrostatic forces occur between the positively charged amino groups in the PCB side chains, which results in a strong expansion of the PCB/PSB IPN hydrogel. Upon pH increase, –COOH groups are gradually deprotonating, which recovers the zwitterionic nature of the PCB side chains and increases the number of the newly formed dipole-dipole zip clusters. As a result, the PCB/PSB IPN hydrogel shrinks reaching a plateau at its SR at pH > 6 (Figure 3). It is interesting to note that the single PCB networks show similar behavior although their SR changes stronger upon pH increase, and the plateau there is observed for pH > 9 [19].

Thus, it could be concluded that each of the PCB/PSB IPN constituents is imparting its own “smart” behavior, defining in this way its dual stimuli responsiveness. These results also confirm that the IPN approach is an effective tool to create smart materials able to respond to more than one external stimulus change.

### 2.5. Salt Responsive Swelling Behaviour of PCB/PSB IPN Hydrogel

**Figure 4:**
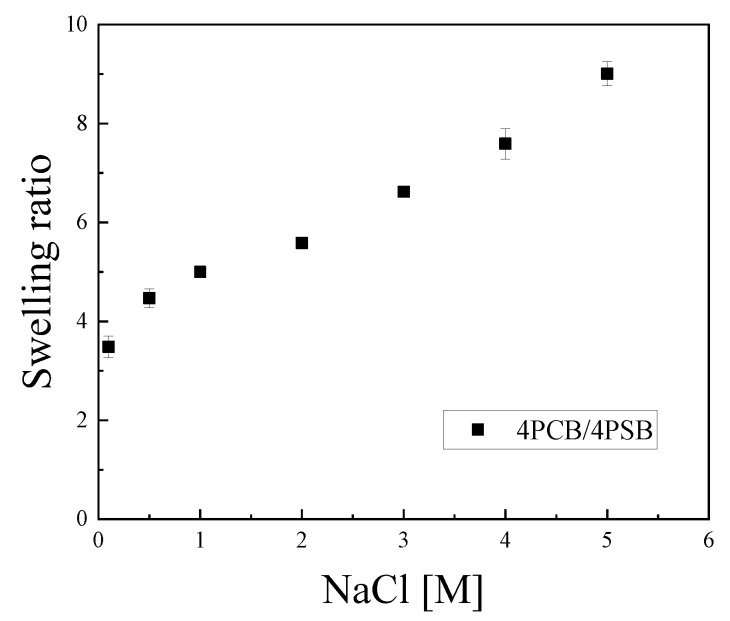
Swelling ratio of PCB/PSB IPN hydrogel in NaCl aqueous solutions with different concentrations at 25 °C.

Since both constituent networks, PSB and PCB, are able to respond to salt concentration changes, exhibiting antipolyelectrolyte behavior, it was expected that their IPN will also possess salt responsiveness. That is why the effect of NaCl concentration on the swelling response of the PCB/PSB IPN network in an aqueous solution was studied (Figure 4). It is seen that the swelling ratio of the IPN hydrogel increases with NaCl concentration increase, i.e., with the ionic strength of the solution increase.

PCB/PSB IPN hydrogel increases 3 times its initial weight when swollen in distilled water, while in NaCl aqueous solutions, with salt concentration ranging from 0.1 M to 5 M, its swelling ratio increases from 3.5 to 9, thus reaching 9 times the increase of its initial weight in the aqueous solution with the highest (5 M) NaCl concentration. This behavior is a result of the shielding effect that the NaCl ions play for the dipole-dipole interactions between the zwitterionic moieties in the IPN [18,19]. The increase in the salt concentration disrupts the interchain dipole-dipole interactions in a way that is akin to the way temperature does it for PSB (Scheme 2) [18]. As a consequence, the IPN hydrogel expands as the NaCl concentration in the aqueous solution increases.

It is interesting to note here that the single PSB [18] and PCB [19] hydrogels show different types of dependence of their swelling ratios with NaCl concentration increase: their SRs increase and level off at NaCl concentrations above 3 M. For the PCB/PSB IPN hydrogel, however, almost a linear dependence of its SR on the NaCl concentration increase is observed. This is a result of the chains interlacing, which occurs between both constituent networks in the IPN, which limits the SR and results in different profiles of the SR’s salt concentration dependence.

The triple stimuli responsiveness of the PCB/PSB IPN hydrogel towards temperature, pH, and salt concentration was convincingly demonstrated so far. We were able to find only one report about a polymer-based triple stimuli-responsive system able to respond simultaneously to changes in temperature, pH, and ionic strength [24]. The system is based on copolymers of *N*-isopropylacrylamide (NIPAM) and acryloyloxyethyl trimethyl ammonium chloride (DAC), reinforced by exfoliated laponite. This material exhibits low critical solution temperature behavior with the ability to collapse upon swelling in aqueous solutions with very low salt concentrations (0.1 M), i.e., exhibiting polyelectrolyte behavior, as well as responding to pH change in a very undefined way (no clear dependence between the pH change and the SR is seen). In this respect, the newly developed PCB/PSB IPN has strongly pronounced responses in terms of its SR change with respect to the three studied stimuli, as well as changes in much wider ranges of their variation. There are also some other studies where triple stimuli-responsive systems to other biological stimuli are reported, e.g., where sensitivity to different biomolecules instead of ionic strength is shown [25,26].

One of the strongest advantages that PZI, and in particular PSB and PCB, possess is their biocompatibility. They are known to be highly biocompatible, which significantly increased the interest in PZI-based materials recently. The PZs’ biocompatibility is related to the very low non-specific protein absorption that the PZIs’ based materials possess, which defines also their antibiofouling activity as well as their good in vivo performance. Thus, the demonstrated improved mechanical performance as well as the triple stimuli responsiveness of the PCB/PSB IPN hydrogel has to be accompanied by a study on their biocompatibility in order to fully reveal their potential as a smart biomaterial.

### 2.6. Freezable and Non-Freezable Water in PCB/PSB IPN Hydrogel

**Figure 5:**
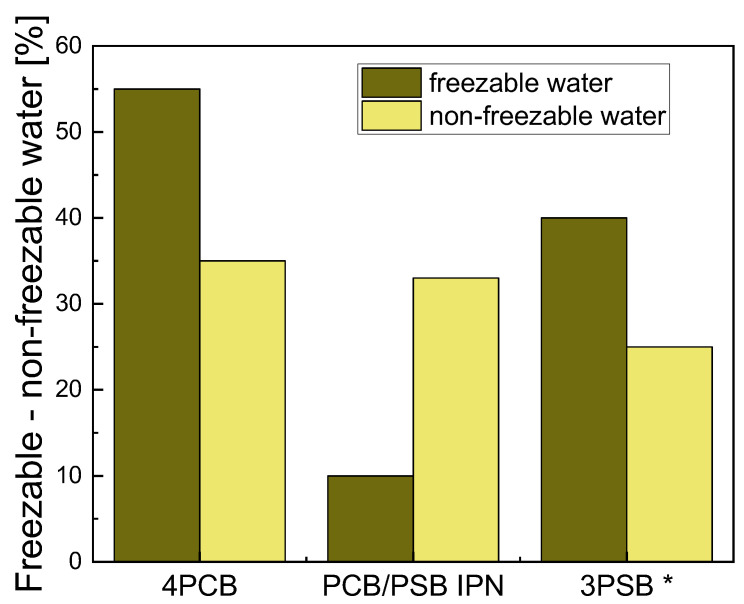
Amount of non-freezable water (NFW) and freezable water (FW) in PCB/PSB IPN hydrogel. For sake of comparison the data for single PSB and PCB networks are also provided [18,19].

The biocompatibility of the PZI-based materials is often explained by the high amount of bound water they are able to retain. Using differential scanning calorimetry (DSC) analysis, the amounts of the non-freezable (NFW) and the freezable (FW) water in PCB/PSB IPN hydrogel were determined (Figure 5). The non-freezable water is considered to be bound by the polymer functional groups, which reduces its mobility and significantly decreases its freezing temperature, i.e., it does not freeze at 0 °C or around. Part of the water molecules in the hydrogel, however, stay “free” from such interactions, and thus they are able to freeze at 0 °C—these are designated as FW. The DSC analysis shows that the amount of FW in PCB/PSB IPN network is 10% while the NFW is ~33%. For the sake of comparison, data for the same two water amounts are provided in Figure 5 for single PCB and PSB networks with the same or comparable crosslinking density.

It is seen in Figure 5 that the PCB/PSB IPN hydrogel has the lowest FW amount and comparable to the single PCB network NFW amount. The low amount of FW could be due to the more entangled structure of the IPN as compared to the single networks, which limit the free water molecules’ diffusion. This explanation is confirmed by the equilibrium swelling ratio (ESR) values for the same samples presented in Table 2, where it is seen that the ESR value of the PCB/PSB IPN is lower than the ESR for the neat single PSB and PCB networks. Nevertheless, applying the above-outlined relation between “bound” NFW water and the biocompatibility, one could expect that the IPN hydrogel would have compared to the single PCB biocompatibility.

It should be mentioned here that PCB is recognized to exhibit better in vivo performance than PSB, as reported by our [18,19] as well as others [27] recent studies.

### 2.7. Bacterial Film Growth Inhibition

PZs are known to exhibit antibiofouling activity, which is explained via their strong interaction with water, which lies behind the formation of a soft hydrated layer on their surface. This hydrated layer makes hard the bacteria attachment and prevents bacterial colonization, thus minimizing the risk of bacterial biofilm formation. To check if the PCB/PSB IPN hydrogel shows such inhibitory activity towards bacterial film growth, we used three commonly spread bacteria, namely *P. Aeruginosa*, *A. Baumanii,* and *K. Pneumoniae*.

*P. Aeruginosa* is a Gram-negative aerobic bacterium that has been identified as a pathogen for humans and plants. It could be found in the environment, e.g., in soil and water, and can cause infections in the blood, lungs (pneumonia), or other parts of the body after surgery.

*K. pneumoniae* is a Gram-negative facultative anaerobic bacterium that although found in the normal flora of the mouth, skin, and intestines, can cause destructive changes to human and animal lungs if aspirated.

*A. Baumannii* is a Gram-negative bacterium that is a pathogen in humans, affecting people with compromised immune systems. It is becoming increasingly important in hospital-derived infection, as it is almost exclusively isolated from hospital environments.

**Figure 6:**
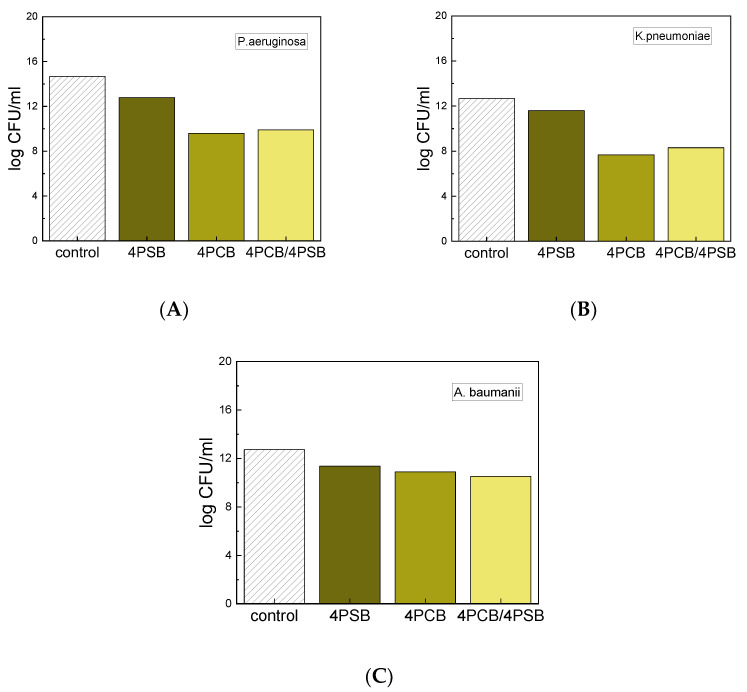
Growth inhibitory activity of 4PSB, 4PCB and PCB/PSB IPN hydrogels towards (**A**) P. Aeruginosa, (**B**) K. Pneumoniae and (**C**) A. Baumanii.

The results demonstrate that PCB/PSB IPN hydrogel reduces the growth of *P. Aeruginosa*, *K. pneumoniae* and *A.baumanii* as compared to the control (Figure 6). It has comparable to the neat 4PCB hydrogel antibiofouling effect towards the three bacteria and has better performance as compared to the neat 4PSB hydrogel. This is additional proof that the IPN approach is able to create novel polymeric materials by utilizing the performance of the constituent single networks. These results also confirm the expected from the FW-NFW study comparable “biological” performance of the PCB/PSB IPN with the one of the PCB single network due to the close NFW amounts they retain.

Biofilm formation usually starts with the formation of conditioning film, which is the biomaterial surface fouled by proteins and other biological compounds present in the body. The conditioning film triggers the cascade of the biofilm formation stages, which include irreversible bacterial attachment, co-adhesion, and extracellular matrix synthesis, followed by microbial colony formation and maturation. The result is a thick biofilm that can disperse planktonic bacteria [28]. The recent efforts in creating antibiofouling materials are devoted to the preparation of surfaces that reduce the non-specific adsorption of proteins and cells, minimizing in this way the conditioning film formation and thus preventing the biofilm formation. The antibiofouling properties are defined by the ability of the materials to form a hydration layer on their surfaces, which is an intrinsic cause for non-fouling surfaces [29]. The tightly bound water molecules form a physical as well as energetic barrier to prevent protein adsorption on the surface. Protein adsorption is known to take place through the expulsion of water molecules from both the surface and the protein in order to reduce the free energy barrier arising from the dehydration entropic effect [29]. The strength of surface hydration is defined by the physicochemical properties of the polymeric materials, e.g., surface chemistry, surface packing (i.e., film thickness, packing density, and chain conformation), etc. In addition to surface hydration, chain flexibility also plays an important role in protein resistance [29]. When protein approaches the polymer surface, the compression of the polymer chains causes steric repulsion to resist protein adsorption due to the unfavorable decrease in entropy. The best non-fouling ability of polymers can only be achieved when surface hydration and steric repulsion work together. Owing to their net charge neutrality, zwitterionic polymers exhibit ultralow protein adsorption (proteins are usually negatively or positively charged) [30]. In addition, zwitterionic polymers are more hydrophilic than poly(ethylene glycol) (PEG), which is the golden standard for non-fouling performance due to their stronger interaction with water molecules via ionic solvation, instead of the hydrogen bonding employed by PEG. Thus, the IPN approach contributes to the steric repulsion of the proteins, additionally enhancing in this way the antifouling properties of the newly developed material.

### 2.8. Cytotoxicity

**Figure 7:**
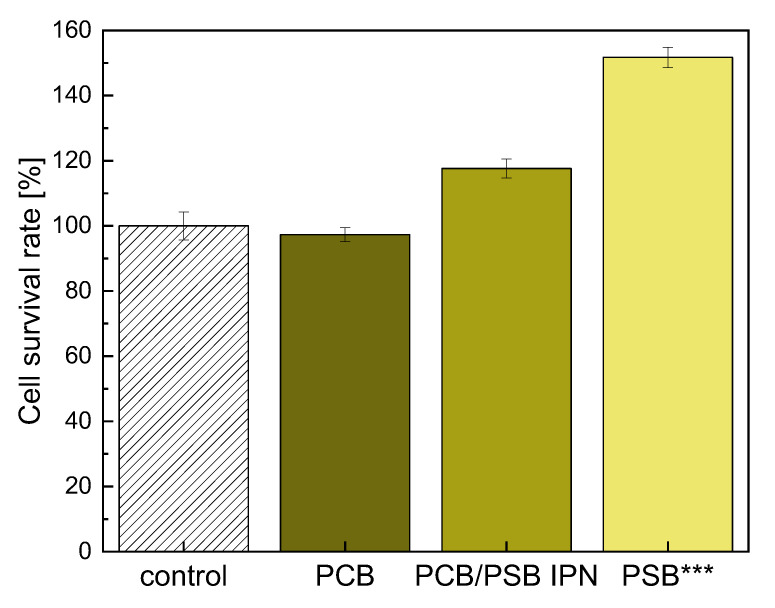
Viability of human embryonic Lep-3 cells seeded on the PCB/PSB hydrogel, assessed with cell proliferation colorimetric assay test (MTS) after 24 h incubation (*** *p* < 0.001 as compared to the control).

Fast-growing human embryonic stem cells (Lep 3) were used to determine the cytotoxicity of the PCB/PSB IPN hydrogel. The Lep 3 cell viability in the presence of the PCB/PSB hydrogel is higher (~117%) as compared to the control (~100%) (Figure 7) after 24 h cultivation. Similar behavior is observed for the 4PSB sample, and thus the behavior of the IPN hydrogels could be explained by the PSB component in its composition. Notably, 4PCB hydrogel at the same time shows comparable to the control cell viability. The results indicate that the PCB/PSB IPN hydrogel is not cytotoxic material and allows cell proliferation—this makes it a suitable candidate for biomedical applications, e.g., for tissue engineering.

### 2.9. In Vivo Biocompatibility Testing

#### 2.9.1. Haematology

The blood analysis of the three studied mice groups demonstrates that the mice from the 1st and the 3rd groups, i.e., the ones where PCB/PSB IPN hydrogels were implanted, have blood parameter counts which are within the reference range of the analyzer’s calibrations. Only the mice with polycon sutures showed white blood cell (WBC) counts at the upper limits of the reference range (Table 3).

Thus, the PCB/PSB IPN hydrogel shows a very good in vivo response compared to the widely used sutures such as the polyamide ones. These results were further confirmed by the histology study.

#### 2.9.2. Histology

**Figure 8:**
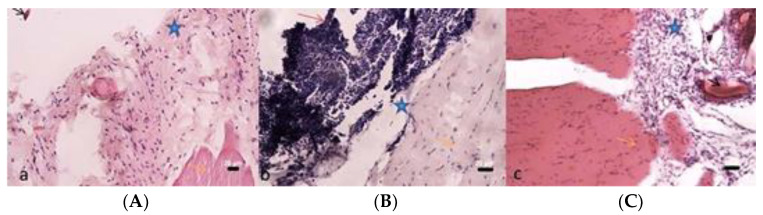
Hematoxylin and eosin-stained sections of the experimental site at the 30th day: (**A**) mouse with implanted hydrogel in the region of the hypodermis and the cutaneous panniculus carnosus muscle; (**B**) mouse with surgical sutures with polycon semi-elastic surgical thread of experimental muscle lacerations in the region of m. biceps femoris and m. semitendinosus; (**C**) mouse with implanted hydrogel in surgical lacerations in the region between m. biceps femoris and m. semitendinosus; Designations: implanted hydrogels (black arrows); fibrous capsule (blue 5-point stars); muscle fibers (orange short arrows); demarcation inflammation (red long arrow). Scale bar 20 µm.

The operative wounds were macroscopically completely healed and hairy on the 30th day of the surgery. During the whole period of study, the mice from all three groups showed good general condition and appetite. The mice from the 2nd and 3rd groups also showed a rapid return of motility in the operated legs. The histological observations showed a very slight reaction in the 1st and the 3rd groups, i.e., the mice where PCB/PSB IPN hydrogels were implanted, namely fibrous capsule formation and absence of inflammatory cells as lymphocytes, plasmocytes, polymorphonuclear leukocytes or macrophages (Figure 8A,C). No signs of immune cell reaction as a part of the foreign body response were found at the 30th day in these groups. In contrast, the 2nd group with resolvable threads had marked infiltration with polymorphonuclear leukocytes and lymphocytes (Figure 8B).

In all three mice groups, a fibrous capsule was formed around either the implanted IPN hydrogels or the surgical polycon node. It was more pronounced in the 1st and the 3rd groups and very thin in the 2nd group, where abundant lymphocytes were presented. On the 30th day, capsulation-linked small blood capillaries in the 1st and the 3rd groups were noticed and the nearby muscles were morphologically preserved—no inflammation, hyalinization of fibers, or atrophy was noticed. According to the observations, the local tissue application of the PCB/PSB IPN hydrogel led to normal activation and duration of the wound-healing process of skin and muscles and preserved leg motility with moderate fibrotic tissue formation. No signs of inflammation, necrosis, calcification, or other pathologies were found when PCB/PSB IPN hydrogel was implanted.

Thus, the in vivo studies on the PCB/PSB IPN hydrogel behavior showed blood parameters within the reference range, capsule formation, normal wound healing process and even small blood capillaries formation around the 30th day without any adverse effects on the skin and muscles around the implantation site. These experiments proved the good in vivo biocompatibility of the PCB/PSB IPN hydrogel. The in vivo studies of PSB [18] and PCB [19] single networks hydrogels showed that the PCB/PSB IPN hydrogel is more effective and has better in vivo performance as compared to them. The implantation of PSB hydrogel resulted in a slight to moderate inflammatory reaction [18], while when the PCB hydrogel was implanted, only single lymphocytes were seen [19]. In contrast, such an inflammatory reaction to the implanted PCB/PSB IPN hydrogel was absent, and they exhibited better biocompatibility. Similar conclusions could be drawn for the foreign body reaction—for PSB a few macrophages were observed in the implantation site [18], which were scarcely seen in the case of PCB [19] and absent in the case of the PCB/PSB IPN.

## 3. Conclusions

In this study, zwitterionic IPN based on PCB and PSB was synthesized and its smart response to changes in three different environmental parameters was demonstrated. Most of the smart materials reported so far are usually able to respond to changes in one or two external stimuli, and thus the development of material that is responsive to three “biological” parameters is unique and opens a wide area for different applications. The study has also demonstrated the potential and versatility of the IPN approach as a method for creating smart materials. The newly synthesized PCB/PSB IPN successfully inherits the smart properties of both constituent single networks and simultaneously responds to variations in temperature, pH, and salt concentration. The PCB component imparts the novel PCB/PSB IPN pH sensitivity as well as biocompatibility, demonstrated by antibiofouling activity, non-cytotoxicity, and very good in vivo performance, while the PSB component imparts temperature responsiveness. Both components, PCB and PSB define the antipolyelectrolyte behavior of the IPN; however, the IPN exhibits its own unique way of salt concentration dependence, which is linear in contrast to the single PSB and PCB salt responsiveness which SRs level off at higher salt concentrations. The PCB/PSB IPN hydrogel exhibits antibiofouling activity against some commonly spread bacteria such as *P. Aeruginosa*, *A. Baumanii* and *K. Pneumoniae,* it is non-cytotoxic and possesses very good in vivo biocompatibility which makes it a unique smart material that can find many applications in different areas, including medicine, pharmacy, smart materials for sensors, etc.

## 4. Materials and Methods

### 4.1. Materials

[2-(Methacryloyloxy)ethyl]dimethyl-(3-sulfopropyl)ammonium hydroxide (sulfobetaine methacrylate, SBMA) (97%), sodium metabisulfite (Na_2_S_2_O_5_, 97%), ammonium persulfate ((NH_4_)_2_S_2_O_8_, (98%), poly(ethylene glycol) diacrylate (PEGDA, average Mn = 575), 2-(dimethylamino) ethyl methacrylate (DMAEMA), tryptic soy broth (TSB), Dulbecco’s Modified Eagle’s Medium (DMEM), tripsin, penicillin, streptomycin, ethylenediaminetetraacetic acid (EDTA), thiazolyl blue tetrazolium bromide powder, dimethyl sulfoxide (99%), phosphate-buffered saline (PBS), mueller hinton brod, mueller hinton agar and bovine serum albumin (BSA), phosphoric acid (H_3_PO_4_), sodium hydroxide (NaOH), sodium chloride (NaCl) were purchased from Sigma-Aldrich (Saint Louis, MO, USA). Ethylmethylketon (≥99%) was purchased from Fluka (Buchs, Switzerland). Beta-propiolactone (97%) was obtained from Alfa Aesar (Haverhill, MA, USA). Ethylene glycol (anhydrous 99.8%) and ethanol were purchased from Laborchemie Apolda (Apolda, Germany). Potassium persulfate (K_2_S_2_O_8_, ≥99%) was purchased from Chem Lab (Zedelgem, Belgium).

Cell proliferation colorimetric assay kit (MTS) was purchased by Abcam (Cambridge, UK)

Zoletil 50 (tiletamine HCl 125 mg, zolazepam base 125 mg) was purchased from Virbac (Carros, France). Xylazine 2% was obtained from Alfasan (Woerden, Netherlands). Butomidor (Butorphanol 10 mg/mL) was bought from Richter Pharma (Wels, Austria).

Klebsiella pneumonia (K. pneumonia) (ATCC 13883), Pseudomonas aeruginosa (P. Aeruginosa) (ATCC 10145), Acinetobacter baumannii (A. Baumanii) (ATCC 19606) were obtained from American Type Culture Collection ATCC (Manassas, VA, USA).

Human embryonic stem Lep3 cells were obtained from the Laboratory of Cell Cultures, National Centre of Infectious and Parasitic Diseases (Sofia, Bulgaria).

### 4.2. Methods

#### 4.2.1. Synthesis of PCB/PSB IPNs

**Scheme 3:**
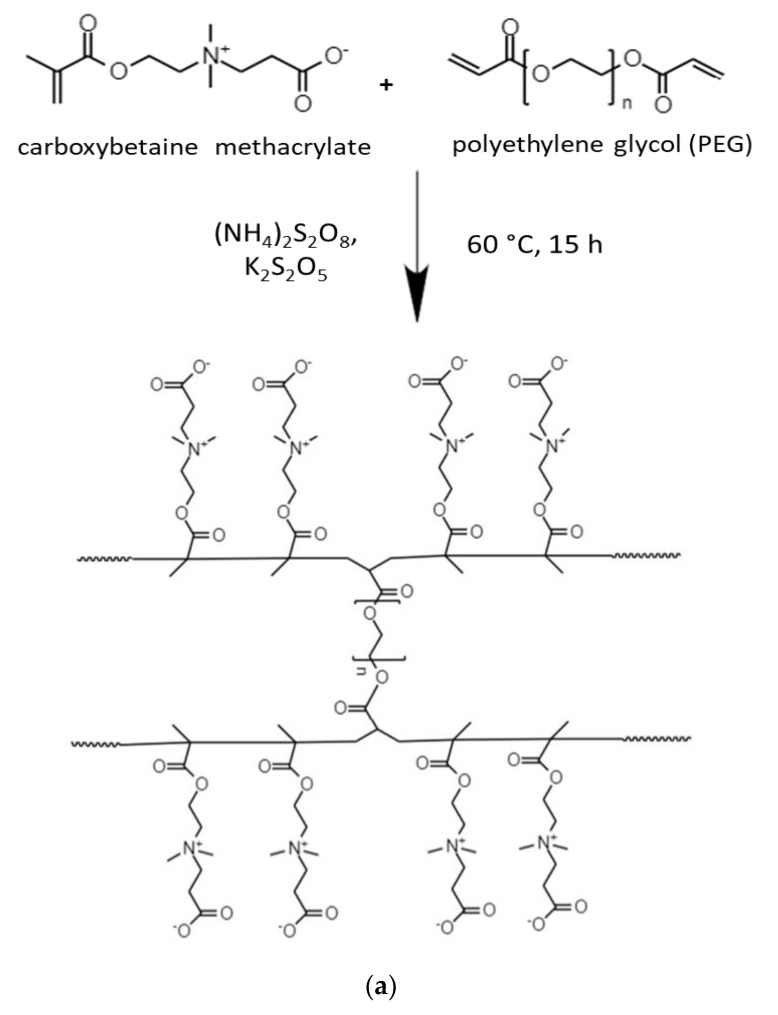

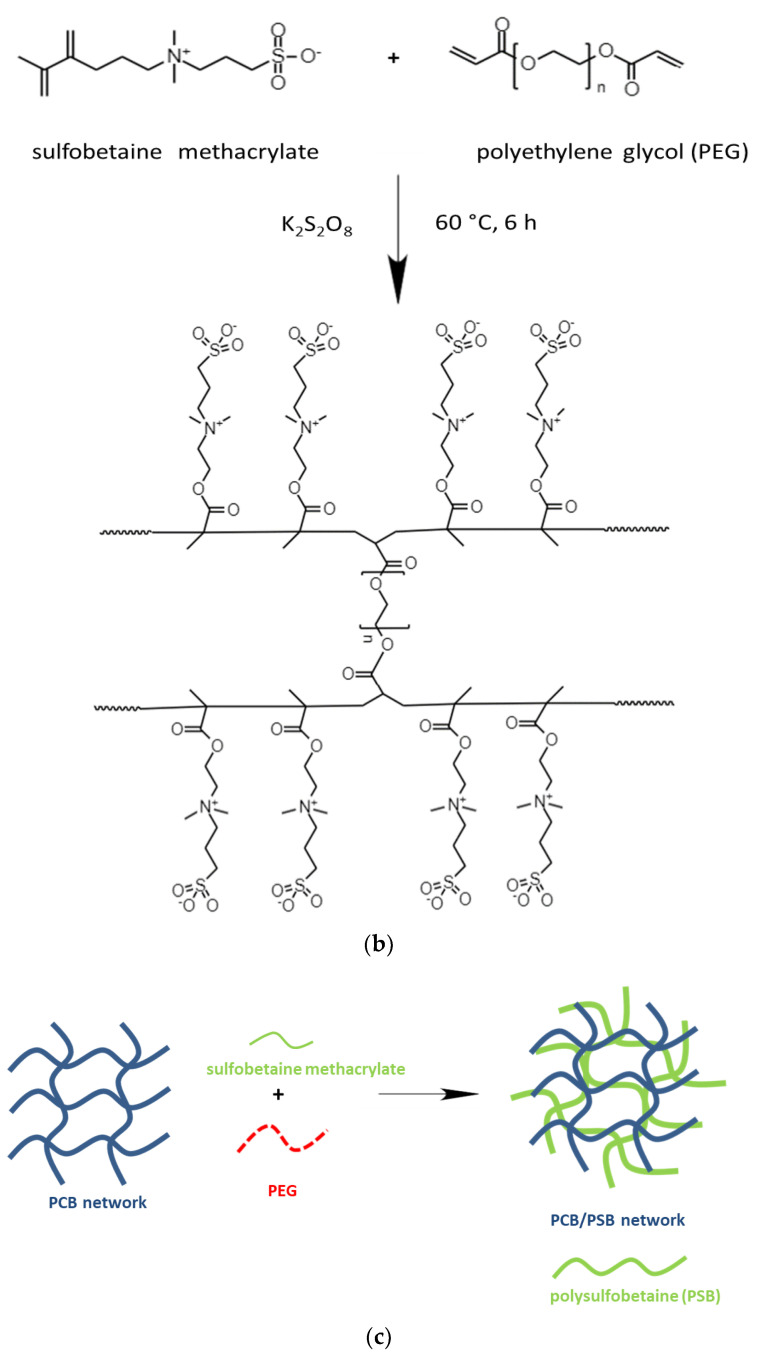
Formation of PCB/PSB IPN by using the sequential method for IPN preparation: (**a**) PCB SN; (**b**) PSB SN and (**c**) sequential approach for PCB/PSB IPN formation.

PCB/PSB IPNs were prepared in two steps via the sequential method using free radical crosslinking polymerization (Scheme 3). To this aim, the monomer carboxybetaine methacrylate (CBMA) was synthesized as described elsewhere [20] and used for the synthesis of the 1st single network, namely PCB. Briefly, 2.30 g CBMA was dissolved in 10 mL mixed solvent of ethylene glycol/ethanol/H_2_O (6:2:2 volume ratio) under constant stirring at 25 °C obtaining in this way 1 M CBMA monomer solution. Notably, 4 mol.% crosslinking agent PEGDA and the initiators Na_2_S_2_O_5_ (0.665 mol.%) and (NH_4_)_2_S_2_O_8_ (1.445 mol.%) were added to the CBMA solution (Scheme 3a). The amounts of the crosslinking agent and the initiators were calculated with respect to the monomer CBMA. After the complete dissolution of all reagents, the solution was poured between two glass plates separated by a rubber spacer and left to polymerize at 60 °C for 15 h. The obtained PCB network was immersed in a large amount of distilled water in order to remove all non-reacted reagents. Water was changed daily and checked via UV-vis spectrophotometer until no unreacted chemicals were detected. The purified PCB hydrogel was finally left to dry at room temperature.

The dry PCB network was subsequently immersed in an excess amount of 1 M SBMA aqueous solution also containing 4 mol.% PEGDA and 0.01 mol.% K_2_S_2_O_8_ and then left at 5 °C until reaching equilibrium swelling (Scheme 3c). The second polymer network, i.e., PSB, was synthesized in situ in the presence of the 1st one, PCB, at 60 °C for 6 h thus obtaining the PCB/PSB IPN (Scheme 3b). The obtained IPN was purified from non-reacted reagents in the same manner as the single PCB.

#### 4.2.2. Characterization of PCB/PSB IPN

##### Microhardness

The Vickers microhardness (Hʋ) of PCB/PSB IPN was measured by using Vickers diamond pyramidal indenter having a square base and a pyramidal angle of 136°, attached to a Leica VMHT universal research microscope (Mannheim, Germany). Three different loads were used and at least 10 measurements were performed with one load on different parts of the surface of the dry PCB/PSB network at 25 °C. Each indentation lasted 16 s. The final Hʋ values were calculated by using the following equation:

Hν = (1.8544P)/d^2^  (kg.mm^−2^) (1)

where P is the load (kgf) and d is the diagonal of the indentation mark (mm) obtained on the IPN’s surface after removing the indenter.

##### Rheology

The mechanical properties of PCB/PSB IPN were studied by using a dynamic rheometer HAAKE RheoStress 600 (Waltham, MA, USA), equipped with a parallel plate sensor system and Peltier temperature controller. Dry networks were left to reach their ESR in distilled water for 3 days and then were cut into disks with 20 mm diameter. Their dynamic storage (G′) and loss (G″) moduli were measured in the 0.1 ÷ 10 Hz frequency range at 25 °C in CD-mode (γ = 0.005).

##### Temperature Responsiveness of PCB/PSB IPN Hydrogels

The ability of PCB/PSB IPN hydrogels to change their swelling ratio (SR) as a function of temperature was investigated in the temperature range of 5 ÷ 85 °C. Dry disk-shaped pieces with weight of ~0.1 g were incubated in 100 mL distilled water (DW) at the subsequent temperature for 8 h. After being taken out from the aqueous medium, the swollen hydrogels were wiped off using fiber-free paper and weighted. Their SR was calculated using the following equation:

SR = (m_s_−m_d_)/m_d_ (2)

where m_s_ is the weight of the swollen hydrogel and m_d_ is the weight of the dry network. The temperature of the aqueous medium was gradually increased by 5 °C for each subsequent measurement and the SR of PCB/PSB IPN hydrogel was determined at each subsequent temperature in the same manner as described above. The SR measurements at each temperature were conducted for at least three different IPN pieces and then averaged.

##### pH Responsiveness of PCB/PSB IPN

Dry disk-shape PCB/PSB IPN samples (~0.1 g) were immersed in 100 mL water with defined pH adjusted by adding either H_3_PO_4_ or NaOH at 25 °C for 72 h until reaching their ESR. The obtained hydrogel was weighted after removing the residual water from its surface by whipping it with fibre-free paper and its ESR was determined using equation (2). The pH dependence of IPN’s swelling was studied in the pH range from 2 to 10. The ESR measurements at each pH were conducted for at least three different IPN pieces and then averaged.

##### Salt Responsiveness of PCB/PSB IPN

Aqueous solutions of NaCl with concentrations ranging from 0.1 M to 5 M were used to study the ability of PCB/PSB IPN to respond to variations in the salt concentration. Dry disk-shape samples form PCB/PSB IPN (~0.1 g) were left to reach their ESR in 0.1 M NaCl solution at 25 °C for 72 h. They were wiped using filter paper and weighted in order to determine their ESR using equation 2. The same hydrogels were then transferred to the next salt solution with higher concentration and the same procedure was repeated. All results are obtained after averaging the ESR values obtained after three independent measurements of three different IPN pieces.

##### Freezable and Non-Freezable Water in PCB/PSB IPN

Differential scanning calorimetry (DSC) was used to analyze the state of water within the IPNs. To this purpose, dry PCB/PSB IPN was let to swell in distilled water at 25 °C for 72 h. Then, a piece from the swollen IPN sample (~6 mg) was sealed in a DSC T zero aluminum pan and analyzed against an empty pan (control) via Q200 Differential Scanning Calorimeter (TA instruments, USA). Both pans were first cooled to −90 °C and then heated to 160 °C at a heating rate of 10 °C/min. A constant flow of 50 mL/min N_2_ gas was purged through the system during the DSC analysis. The amounts of freezable (W_fs_) and non-freezable (W_nfs_) water were calculated using the following equations [31]:

SR [%] = ((m_s_ − m_d_)/m_s_)*100 (3)

W_fs_ [%] = (ΔH_hydroge_l/ΔH ^ͦ^ _H2O_)*100 (4)

W_nfs_ [%] = W_s_ − W_fs_ (5)

where SR is the swelling ratio of IPN hydrogel, m_s_ is the weight of the IPN hydrogel after 72 h swelling into the salt solution with respective concentration m_d_ is weight of the dry network; ΔH°_H2O_ = 333.5 J/g is the water melting enthalpy and; ΔH_hydrogel_ is the sum of the melting enthalpies for all endothermic peaks which appear in the DSC heating run; W_fs_ is the freezable/unbonded water and W_nfs_ is the non-freezable/bonded water.

##### Bacterial Growth Inhibition

The bacterial attachment and biofilm formation assay was performed in sterile Eppendorf tubes. Three different bacterial strains were used, namely *K. pneumoniae* (ATCC 13883), *P.aeruginosa* (ATCC 10145), and A. baumanii (ATCC 19606). Each strain was grown overnight in Mueller Hinton Broth at 150 rpm and 37 °C. The obtained bacterial cultures were diluted with physiological solution to match the turbidity of a 0.5 McFarland standard (approximate cell density 1.5 × 10^8^ mL). Dry PCB/PSB IPN samples (~0.1 g), sterilized in ethylene oxide, were placed in an Eppendorf tube containing 2 mL bacterial inoculum. Bacterial inoculum without hydrogel was used as a control. All Eppendorf tubes were left at 37 °C for 24 h. Thereafter, the liquid was gently removed and the hydrogels in the tubes were washed twice with freshly prepared PSB in order to remove the loosely attached bacteria. For enumeration of bacteria, 1 mL of physiological solution was added to each Eppendorf tube followed by ultrasound sonication for 20 min. Several dilutions were completed prior to plate the bacterial suspension on Muller Hinton agar plates. After 24 h at 37 °C, the number of colonies on the agar plates was counted to determine the concentration of live bacterial cells.

##### Cytotoxicity

The cell viability of human embryonic fibroblasts Lep-3 when in a contact with PCB/PSB IPN was assessed by MTS test according to the manufacturer’s instructions. The cells were grown as monolayer cultures in Dulbecco’s modified Eagle’s medium (DMEM), supplemented respectively with 10% fetal bovine serum, 100 U mL^−1^ penicillin, and 100 μg mL^−1^ streptomycin at 37 °C in a humidified CO_2_ incubator (Thermo Scientific, Hepa class 100). For routine passages, the cells were detached using a mixture of 0.05% trypsin and 0.02% ethylenediamine tetra acetic acid (EDTA).

The MTS test was performed as the dry PCB/PSB networks were first swollen in sterilized distilled water for 24 h and subsequently soaked in DMEM medium for several hours. Then, they were cut into small uniform pieces and transferred into a 24-well tissue culture plate where they were sterilized by UV radiation for 2 h. A cell suspension with a density of 7.5 × 10^4^ cells/well was added to the plate. The cell-seeded hydrogels were maintained at 37 °C under 5% CO_2_ for 24 h, after which the 100 µL MTS reagent was added to each well. Finally, the optical density of the obtained solution was measured at 540 nm. As a control, a suspension of cells grown in non-modified medium without the presence of PCB/PSB IPN hydrogel was used. The cell viability (%) in the presence of PCB/PSB IPN hydrogels was determined relative to the cell viability found in the control. All results are reported as the mean ± standard error of the mean values obtained from three independent measurements determined using one-way analysis of variance (ANOVA).

#### 4.2.3. In vivo Biocompatibility Test. Animal Design and Implantation of Biomaterials

##### Mouse Model

The in vivo experiments were consistent with the regulations of local institutional, Bulgarian National Regulation № 20/01.11.2012 regarding laboratory animals and animal welfare and European legislation.

In the in vivo experiments were used 12 male albino laboratory mice, 4-month-old weighting about 25 g each. They were divided into 3 groups:

- 1st group—mice with dorsal subcutaneous implantation of PCB/PSB IPN hydrogel with 2–3 mm in diameter
- 2nd group—mice with experimentally made muscle lacerations in the region of m. biceps femoris and m. semitendinosus and surgical sutures with polycon semi-elastic surgical thread
- 3rd group—mice with implantation of PCB/PSB IPN hydrogel pieces which are 2–3 mm in diameter in the region of fossa poplitea between m. biceps femoris and m. semitendinosus.

After 4 week evaluation period, all animals were humanely euthanized and materials for histology and hematology were taken.

##### Rodent Injectable Anesthesia

General anesthesia was made using atropine (0.04 mg/kg) as premedication and anesthesia mixture of tiletamine/zolazepam, xylazine and butorphanol and standard sterilization with ethyl alcohol and braunol solution, after shaving of the hair. Preparation of anesthesia: in a vial powder Zoletil 50 (tiletamine HCl 125 mg, zolazepam base 125 mg) is administrated 10 mL Xylazine 2% and 0.75 mL Butomidor (Butorphanol 10 mg/mL) in doses of 0.1–0.2 mL/kg.

##### Surgical Procedures

Incorporation of the hydrogels in PBS was carried out after sterilization with ultraviolet irradiation (UV) for 4 h and aseptical conditions. Standard surgical access through the skin-dermis and hypodermis in the dorsal region in the area of the withers (in the 1st group) and to fossa poplitea between biceps femoris and semitendinosus muscles in the 2nd and 3rd groups was made. Implants were situated in the 1st and 3rd groups and tissues were then surgically restored, in the 2nd group were made several polycone sutures and nodes for comparison of the local tissue reaction.

##### Hematology

Blood samples from each experimental groups were taken in sterile vacuum blood collection tubes via cardiac puncture. Hematological analyses were performed by automatic xematology analyzer “Mindray BC—2800 Vet”, Mainland China. The values of the counts of routine blood parameters were checked in all groups.

##### Histology

Tissue samples (0.6 cm^3^ in size) with incorporated implants or sutures were routinely fixed in 10% buffered formalin, dehydrated in ethanol, and embedded in paraffin.

Tissue samples (0.6 cm^3^ in size) with incorporated implants or sutures were routinely fixed in 10% buffered formalin, dehydrated in ethanol, and embedded in paraffin. Tissue sections (3–5 µm thick) were stained in hematoxylin and eosin and examined by light microscope (Leica DM 5000B, Wetzlar, Germany). The sections were scored for the presence of fibrotic tissues, vascularization, inflammatory cell reactions, and foreign body response.

## Figures and Tables

**Table 1:** Microhardness of dry PCB/PSB IPN, single 4PSB and 4PCB networks.

Sample	Microhardness [Mpa]
4PCB *	86.7 ± 3.2
**PCB/PSB IPN**	14.6 ± 1.1
4PSB *	27.2 ± 2.7

* The microhardness values of 4PSB and 4PCB are taken from [23].

**Table 2:** ESR of PCB/PSB IPN hydrogel in distilled water. For sake of comparison the data for single PSB and PCB networks are also provided [18,19].

	4PSB	PCB/PSB IPN	3PSB *
**ESR**	7.5^18^	2.9	4.1^19^

* 3PSB is the closest to 4PSB single PSB network, for which we have such data, it is obtained by using 3 mol.% PEGDA crosslinking agent.

**Table 3:** Haematological data taken from male mice, representative of the 2nd group.

Parameter	Data	Range
White blood cells count (WBC)	14.4 × 10^9^/L	5.05–15.76 × 10^9^/L
Lymphocytes count (Lymph)	11 × 10^9^/L	3.20–11.02 × 10^9^/L
Monocytes count (Mon)	0.3 × 10^9^/L	0–0.43 × 10^9^/L
Granulocytes count (Gran)	3.1 × 10^9^/L	0.75–3.51 × 10^9^/L
Red blood cells count (RBC)	7.1 × 10^12^/L	6.66–10.52 × 10^12^/L
Hemoglobin (HGB)	14 g/dL	13.9 to 15.9 g/dL
Hematocrit (HCT)	39.60%	39–49%
Mean corpuscular volume (MCV)	43.5 fL	41.2–48.4 fL
Mean corpuscular hemoglobin (MCH)	16 pg	14.1–17.1 pg
Mean corpuscular hemoglobin count (MCHC)	340 g/L	328–357 g/L
Red cell distribution width (RDW)	19%	18.8–19%
Platelets count (PLT)	557 × 10^9^/L	402–1098 × 10^9^/L
Mean platelet volume (MPV)	6.4 fL	4.3–8.07 fL

## Data Availability

The raw/processed data required to reproduce these findings cannot be shared at this time as the data also form part of an ongoing study.

## Abbreviations

IPN	interpenetrating polymer network
P. Aeruginosa	Pseudomonas aeruginosa
A. Baumanii	Acinetobacter baumannii
K. Pneumoniae	Klebsiella pneumonia
IPNs	interpenetrating polymer networks
PZs	polyzwitterions
PSB	polysulfobetaines
PCB	polycarboxybetaines
PPhB	polyphosphobetaines
G′	storage moduli
G″	loss moduli
SR	swelling ratio
PEGDA	poly(ethylene glycol) diacrylate
NIPAM	N-isopropylacrylamide
DAC	acryloyloxyethyl trimethyl ammonium chloride
DSC	differential scanning calorimetry
NFW	non-freezable water
FW	freezable water
ESR	equilibrium swelling ratio
PEG	poly(ethylene glycol)
Lep 3	human embryonic stem cells
MTS	cell proliferation colorimetric assay test
WBC	white blood cell
CBMA	carboxybetaine methacrylate
Na_2_S_2_O_5_	sodium metabisulfite
(NH_4_)_2_S_2_O_8_	ammonium persulfate
K_2_S_2_O_8_	potassium persulfate
Hʋ	Vickers microhardness
DW	distilled water
m_s_	weight of the swollen hydrogel
m_d_	weight of the dry network
H_3_PO_4_	phosphoric acid
NaOH	sodium hydroxide
NaCl	sodium chloride
W_fs_	amounts of freezable water
W_nfs_	amount of non-freezable water
ΔH°_H2O_	water melting enthalpy
ΔH_hydrogel_	the sum of the melting enthalpies for all endothermic peaks in the DSC heating run
DMEM	Dulbecco’s modified Eagle’s medium
EDTA	ethylenediamine tetra acetic acid
UV	ultraviolet irradiation
